# Editorial: Advances in multi-modality imaging for precision medicine in cardiomyopathies

**DOI:** 10.3389/fcvm.2025.1650448

**Published:** 2025-07-10

**Authors:** Malgorzata Wamil, Emmanuel Androulakis

**Affiliations:** ^1^ Cardiology Department, Mayo Clinic Healthcare, London, United Kingdom; ^2^ Cardiology Department, Great Western Hospital, Swindon, United Kingdom; ^3^ Deep Medicine Nuffield Department of Women's and Reproductive Health, University of Oxford, Oxford, United Kingdom; ^4^ Cardiology Department, King’s College London, London, United Kingdom

**Keywords:** cardiomyopathy, hypertrophic cardiomyopathy, dilated cardiomyopathy, cardiac MRI, precision medicine, imaging

**Editorial on the Research Topic**
Advances in multi-modality imaging for precision medicine in cardiomyopathies

Inherited and acquired cardiomyopathies encompass a diverse range of heart muscle diseases. In recent years, significant advances in cardiovascular imaging techniques have enabled more precise diagnosis and risk assessment. The 2023 European Society of Cardiology (ESC) guidelines for cardiomyopathies explicitly endorse a multimodality imaging approach to achieve more accurate phenotyping and patient-tailored care. (1) Within this Frontiers Research Topic, four studies illustrate the power of advanced multimodality imaging in enhancing precision medicine for cardiomyopathy patients. Here, we summarise their findings and reflect on broader implications for clinical practice and future research.

## Imaging-based risk stratification in childhood dilated cardiomyopathy

Street-de Palma et al. present a timely synthesis of imaging-derived prognostic markers in childhood dilated cardiomyopathy (DCM), where evidence has lagged behind adult populations. Their meta-analysis highlights left ventricular dilatation, impaired systolic function, and severe mitral regurgitation as consistent predictors of adverse outcomes. While these markers intuitively mirror risk factors in adult DCM, the findings reinforce the applicability of standard echocardiographic indices in paediatric risk stratification. Notably, this cohort's limited prognostic utility of late gadolinium enhancement underscores the need for age-specific validation of advanced imaging markers. The study illustrates the importance of structured imaging follow-up and underscores the pressing need for prospective multicenter datasets to refine paediatric-specific risk models within a precision medicine framework.

## CMR-derived hemodynamic forces in diabetic cardiomyopathy

Shao et al. introduced hemodynamic force (HDF) analysis as a novel CMR-derived marker for detecting early myocardial dysfunction in asymptomatic type 2 diabetes mellitus (T2DM). Despite having preserved ejection fraction and normal strain on conventional imaging, diabetic patients exhibited abnormal HDF patterns, particularly elevated transversal forces, which were associated with disease duration, myocardial fibrosis, and insulin use. These findings highlight HDF as a potentially sensitive early biomarker that precedes traditional measures of dysfunction. Importantly, it also underscores the promise of integrating functional imaging and metabolic profiling to support preventive, personalised care in T2DM-related cardiac disease.

## Tissue sodium imaging in heart failure phenotypes

Hashemi et al. explore 23Na-MRI as a novel tool to assess myocardial and systemic sodium content across heart failure (HF) phenotypes. In this small proof-of-concept study, 29 patients with HFpEF, HFmrEF, and HFrEF underwent sodium imaging to evaluate clinical severity. While no significant differences in myocardial or skin sodium levels were found across HF subtypes or NYHA classes, subtle trends were noted within the HFrEF group, where higher sodium content correlated with NT-proBNP and impaired renal function, surrogates of fluid overload. Although promising, these findings must be interpreted cautiously due to the limited sample size, phenotypic heterogeneity, and lack of power to detect subgroup differences. Nevertheless, this feasibility study provides early evidence that tissue sodium imaging may complement conventional assessments by capturing subclinical volume status and guiding tailored diuretic strategies.

## Combined genotype and scar imaging to refine risk prediction in hypertrophic cardiomyopathy

Zhang et al. demonstrated the additive prognostic value of late gadolinium enhancement (LGE) on cardiac MRI and genotype status in patients with hypertrophic cardiomyopathy (HCM). This study reinforces the synergistic value of combining advanced imaging markers with genetic profiling for risk stratification in hypertrophic cardiomyopathy (HCM). While LGE and genotype have been individually linked to adverse outcomes, this work offers a novel stratification framework by integrating both modalities. Although retrospective and limited by cohort size, these findings support the utility of genotype–phenotype integration for refining risk assessment and targeting interventions such as ICD placement.

## CMR differentiation of hypertrophic phenotypes for precision diagnosis

Among the studies in this collection, the work by Zhao et al. arguably carries the most immediate clinical impact, addressing a long-standing and diagnostically consequential challenge: distinguishing HCM from hypertensive heart disease (HHD). In this meta-analysis of 26 studies encompassing over 2,900 patients, authors demonstrated that advanced CMR parameters, including native T1 mapping, extracellular volume (ECV), wall thickness, and chamber volumes, differentiate these two hypertrophic phenotypes. Their findings reflect fundamental differences in pathophysiology: HCM, as a primary sarcomeric cardiomyopathy, is marked by more pronounced hypertrophy, diffuse and focal fibrosis, and preserved chamber size, while HHD reflects adaptive remodelling with comparatively lower fibrosis and larger ventricular volumes. Misclassification between HCM and HHD risks undertreatment or overinvestigation, particularly in the context of family screening, genetic counselling, and sudden death prevention. Zhao et al.'s study reinforces that native T1 and LGE, key tissue characterisation tools, can anchor a more accurate, imaging-driven diagnosis. Importantly, the authors call for greater standardisation of CMR acquisition and reporting, a critical next step if these metrics are to be integrated into diagnostic algorithms.

## Translating advancements in imaging into practice: limitations and opportunities

Despite its transformative potential, integrating multi-modality imaging into routine cardiomyopathy care faces significant challenges. A central issue is the limited generalisability of current evidence. As the paediatric DCM and HCM-HHD meta-analyses highlighted, many imaging biomarkers are derived from small, retrospective, or heterogeneous cohorts. Prospective multicentre studies are needed to validate thresholds and to determine how imaging should modify clinical decision-making. Even well-established markers such as LGE or strain lack universally accepted quantitative cut-offs for intervention.

While conceptually compelling, novel techniques like hemodynamic force (HDF) analysis or tissue sodium imaging remain confined mainly to specialised centres. Their clinical translation will depend on technical standardisation, simplified protocols, and demonstration of incremental value over existing methods. Moreover, resource constraints remain a barrier to implementation, particularly in low- and middle-income settings where access to CMR or advanced echocardiographic analysis is limited. Interpreting advanced imaging findings, such as T1 mapping or diastolic strain, also requires expert-level proficiency, raising concerns about equity and consistency in care delivery.

An added layer of complexity lies in the integration of multimodal data. Contemporary cardiomyopathy assessment increasingly encompasses echocardiography, cardiovascular magnetic resonance, genetic profiling, and circulating biomarkers. The synthesis of these heterogeneous data into a unified, clinically actionable risk framework remains a significant challenge. Emerging decision-support algorithms, including those leveraging machine learning, promise to harmonise these inputs for personalised risk stratification. However, their clinical utility will depend on robust validation, clinician uptake, and demonstrable impact on outcomes. Economic considerations are also paramount; while serial CMR and advanced imaging may enhance diagnostic precision, their deployment must be judicious to maintain system sustainability. An optimal balance between precision medicine and pragmatic healthcare delivery will be essential.

## Future directions

The four studies featured in this Research Topic illustrate how advanced imaging continues to refine our understanding and management of cardiomyopathies. From prognostic stratification in paediatric DCM to functional assessment in diabetic patients and from differentiating HCM from its mimics to exploratory tissue sodium mapping, these contributions reinforce the role of imaging not only in diagnosis but also as a central pillar of precision medicine. They echo the direction set by contemporary guidelines: that patient-specific, imaging-guided approaches can inform tailored interventions, improve outcomes, and enable earlier disease interception. Crucially, they highlight the following steps: the need for larger, prospective studies, consensus on quantitative imaging thresholds, and the integration of imaging with genetic and molecular data to advance precision risk models. These studies reaffirm that multi-modality imaging is no longer an adjunct but a cornerstone in the evolving paradigm of cardiomyopathy management.
